# Childhood Growth Trajectories According to Combinations of Pregestational Weight Status and Maternal Smoking during Pregnancy: A Multilevel Analysis

**DOI:** 10.1371/journal.pone.0118538

**Published:** 2015-02-13

**Authors:** Kohta Suzuki, Miri Sato, Wei Zheng, Ryoji Shinohara, Hiroshi Yokomichi, Zentaro Yamagata

**Affiliations:** 1 Department of Health Sciences, Interdisciplinary Graduate School of Medicine and Engineering, University of Yamanashi, Chuo, Yamanashi, Japan; 2 Center for Birth Cohort Studies, Interdisciplinary Graduate School of Medicine and Engineering, University of Yamanashi, Chuo, Yamanashi, Japan; Iran University of Medical Sciences, IRAN, ISLAMIC REPUBLIC OF

## Abstract

Pregestational weight status and maternal smoking during pregnancy are significantly associated with fetal and childhood growth. However, few studies have examined associations between childhood growth and combinations of these factors using multilevel analysis. This study aimed to describe differences in childhood growth trajectories according to these combinations, using data from a prospective cohort study in Japan. The study participants were 1,973 women and their singletons, who were born between April 1, 1991 and March 31, 2003. Children were categorized according to whether they were born to normal-weight, nonsmoking mothers (NN); normal-weight, smoking mothers (NS); underweight, nonsmoking mothers (UN); underweight, smoking mothers (US); overweight, nonsmoking mothers (ON); or overweight, smoking mothers (OS). Birth weight and anthropometric data were collected from 1,965 children at birth (99.6%), 1,655 aged 3 (83.9%), 1,527 aged 5 (77.4%), 1,497 aged 7–8 (75.9%), and 1,501 aged 9–10 (76.1%). Multilevel analysis examining both individual and age as different level variables according to sex was used to describe the trajectories of body mass index z scores for statistical analyses. Although children of the OS group were the leanest at birth, their body mass indices had increased rapidly by 3 years of age. Moreover, body mass index was also likely to increase in boys in the NS and ON groups. A different trend was observed in girls. Body mass index decreased from 5 years of age in girls in the US group. There were no remarkable differences in body mass index trajectories between children in the other groups. In conclusion, childhood growth trajectories differed according to combinations of pregestational weight status and maternal smoking during pregnancy. Further, there were sex-related differences in the associations between childhood growth and factor combinations.

## Introduction

Childhood obesity has become a major public health concern globally, as its prevalence has progressively increased [1]. Obesity and obesity-related diseases, such as diabetes and cardiovascular disease, in adulthood are mainly caused by childhood obesity [2–4]. Further, maternal smoking during pregnancy has been identified as an important risk factor for childhood obesity [5–12]. Children whose mothers smoked during pregnancy are likely to be overweight in infancy and childhood because of fast catch-up growth leading to rapid weight gain during early childhood [13]. A sex difference has also been observed in the association between maternal smoking during pregnancy and later growth in childhood [14]. In our previous studies regarding maternal smoking during pregnancy and childhood obesity [11,12], we had obtained anthropometric data for children of various ages; therefore, we used a multilevel analysis, which is appropriate for data with repeated measurements, to describe growth trajectories during childhood. Twisk stated that multilevel analysis is usually suitable for the analysis of correlated data such as ours [15].

Fetal and early childhood environments have also been found to influence later childhood growth and development. These associations are known as “fetal programming,” “Barker’s hypothesis,” and “the developmental origins of health and disease (DOHaD) hypothesis” [16]. For example, some studies have suggested that there could be an association between the development of chronic diseases in adulthood and a specific path of growth, namely slow growth in fetal life and rapidly increasing body mass index (BMI) in infancy [16–20]. Therefore, it is important to consider the effects of fetal environment, which is reportedly affected by maternal habits such as smoking during pregnancy, on later growth.

In addition to maternal smoking during pregnancy, low pregestational BMI is associated with fetal growth restriction [21–23]. In contrast, maternal pregestational obesity has been identified as a risk factor for obesity in children [23].

With respect to the association between maternal body composition and smoking, rates of smoking during pregnancy have been found to be higher in underweight women, relative to other women, in previous studies conducted in Japan and Korea [24,25]. Furthermore, because postpartum smoking relapse has been associated with weight concern [26], maternal smoking during pregnancy could also be associated with maternal weight status. Therefore, maternal weight status and smoking during pregnancy could affect fetal and childhood growth.

However, no studies have been conducted to examine the effects of the interaction between maternal smoking during pregnancy and pregestational weight status on childhood growth. Therefore, examination of the combined effects of maternal smoking during pregnancy and pregestational BMI on childhood growth could be important. The present study aimed at examining the association between a combination of maternal smoking during pregnancy and pregestational BMI and later childhood growth, based on the sex of the child.

## Methods

### Study Design

The study population comprised children born in Koshu City in Yamanashi Prefecture, Japan between April 1, 1991 and March 31, 2003 and their mothers. They participated in Project Koshu (formerly Project Enzan), a dynamic, ongoing prospective cohort study of pregnant women and their children in rural Japan, which commenced in 1988. Details of this project have been described in previous articles [11,12,14].

A questionnaire-based survey was conducted to determine the nature of the lifestyles of expectant mothers who visited the city office to register their pregnancies. Subsequently, at each medical checkup performed for the children, data regarding their growth and physical characteristics were collected; in addition, anthropometric data were collected for elementary school children and measured annually in April according to Japanese school health law.

This study was approved by the ethical review board at the University of Yamanashi School of Medicine and conducted in accordance with the Guidelines Concerning Epidemiological Research (Ministry of Education, Culture, Sports, Science, and Technology and Ministry of Health, Labour and Welfare, Japan), with the cooperation of the Koshu City administration office. We obtained written informed consent from the participants.

### Data Collection

Data concerning maternal smoking status during early pregnancy were obtained from the mothers using a self-report questionnaire at pregnancy registration. In Japan, it is mandatory for expectant mothers to register their pregnancies in order to access health care services during pregnancy. In the study area, over 80% of the expectant mothers registered their pregnancy in the first trimester, and almost all expectant mothers were registered by 18 gestational weeks. Smoking status was categorized dichotomously, that is, the “smoking mother” category only included those participants who answered “smoking,” and the “nonsmoking mother” category included those who answered “have quit smoking” or “have never smoked.”

The height and weight of expectant mothers were measured at the first prenatal checkup, which was usually carried out during or immediately prior to pregnancy registration. We collected these data as pregestational maternal anthropometric data from expectant mothers when they registered their pregnancies at the city office. BMI was used as a parameter for evaluating maternal obesity and was calculated according to World Health Organization (WHO) standards (weight [kg]/height [m^2^]). Values defining underweight, normal-weight, and overweight categories were BMI < 18.5, BMI 18.5–24, and BMI ≥ 25, respectively.

Data regarding birth height and weight, birth order, and gestational week of delivery were obtained from the Maternal and Child Health Handbook. This handbook is an official publication containing guidelines for obstetric professionals and pregnant women. Moreover, pregnant women used a booklet to record their health status during pregnancy in addition to birth outcomes. Data concerning childhood height and body weight were collected via physical measurements taken during medical checkups conducted when the children were aged 3 and 5 years. These parameters were measured again during medical checkups performed when the children attended grades 2 and 4 at elementary school (i.e., aged 7–8 and 9–10 years, respectively). Height was measured using a stadiometer (unit: 0.1 cm), and body weight was measured using conventional weighing scales (unit: 100 g).

### Statistical Analysis

We initially used a chi-square test to assess the association between children’s sex and follow-up rates at each age and determine the proportions of each group. The individual growth analysis method (SAS PROC MIXED) was used to compare childhood BMI z-score trajectories between groups according to sex. We did not exclude the participants for whom data concerning BMI z scores were missing, because SAS PROC MIXED automatically handles missing data using maximum likelihood. Adopting the approach used by Fitzmaurice et al., we used the following model to explore the differences in the slopes for each interval between the ages of measurement, as our previous findings indicated nonlinearity in the slopes for BMI and BMI z scores [27].

BMI z score_it_ = β1 + (β2 * Time_it_ + β3 * Maternal smoking status during pregnancy (Smoking_it_)) + (β4 * Maternal pregestational weight status (Weight_it_)) + (β5 * Smoking_it_ * Weight_it_) + (β6 * Time_it_ * Smoking_it_) + (β7 * Time_it_ * Weight_it_) _+ (_β8 * Time_it_ * Smoking_it_ * Weight_it_) + e_it_ (where i represents individual, t represents time, β1–8 represents estimates, and e is the error term).

In the final models, years were used to form dummy variables for time. Sample clustering within individuals was addressed. In this analysis, individual BMI data recorded at birth and at least once after the children were aged 3 years were used. Individual BMI z scores, which were based on WHO standards, were used to adjust differences in BMI for each month of age within age groups [28].

Thereafter, children were categorized according to whether they were born to normal-weight, nonsmoking mothers (NN); normal-weight, smoking mothers (NS); underweight, nonsmoking mothers (UN); underweight, smoking mothers (US); overweight, nonsmoking mothers (ON); or overweight, smoking mothers (OS). We calculated estimated BMI z scores for each group at each age using the solution from the final model to describe the trajectories. For example, BMI z scores for boys aged 3 years were calculated as follows:

BMI z score at 3 years of age = intercept (β1) + β2 at 3 years of age + β3 of Smoking + β4 of Weight + β5 of interaction term between Smoking and Weight + β6 at 3 years of age and Smoking + β7 of 3 years of age and Weight + β8 of 3 years of age, Smoking, and Weight.

All analyses were conducted using SAS version 9.3 (SAS Institute, Inc., Cary, NC, USA).

## Results

### Participants

During the study period, 2,284 singleton babies were born. Of these, we obtained the complete data for 1,973 (86.4%) babies and their mothers. There were 1,021 (51.8%) boys. There were no significant sex differences between children with respect to follow-up rates for each age measured (Table 1).

**Table 1:** Number of participants with follow-up measurements at each age for boys and girls.

Age	Boys		Girls		p^a^
	(n = 1,021)		(n = 952)		
	N	%	n	%	
Birth	1,015	99.4	950	99.8	0.3
3 years	855	83.7	800	84.3	0.9
5 years	788	77.2	737	77.4	0.9
7–8 years	791	77.5	706	74.2	0.09
9–10 years	792	77.6	709	74.5	0.11

^a^ *P* values were calculated using chi-square or Fisher’s exact tests

Regarding maternal weight status, 357 (18.1%), 1,471 (74.6%), and 145 (7.4%) mothers were categorized as underweight, normal weight, and overweight, respectively. Moreover, smoking rates for underweight, normal-weight, and overweight mothers were 8.1% (29/357), 5.7% (84/1,471), and 11.7% (17/128), respectively. Smoking rates differed significantly according to weight status (p < 0.01). There was no significant difference in the proportions of boys and girls in each group (p = 0.3; Table 2).

**Table 2:** Comparison of numbers of boys and girls in each group.

Variables	Boys		Girls		p^a^
	n	%	n	%	
Normal-weight, nonsmoking mothers (NN)	706	50.9	681	49.1	0.3
Normal-weight, smoking mothers (NS)	52	61.9	32	38.1	
Underweight, nonsmoking mothers (UN)	165	50.3	163	49.7	
Underweight, smoking mothers (US)	17	58.6	12	41.4	
Overweight, nonsmoking mothers (ON)	71	55.5	57	44.5	
Overweight, smoking mothers (OS)	10	58.8	7	41.2	

^a^ *P* values were calculated using chi-square tests

Means of maternal age, maternal BMI, gestational week, birth order, birth weight, and infant BMI at birth are presented in Table 3 and Table 4.

**Table 3:** Characteristics of mothers in each group.

Variables	Maternal age		Maternal BMI^b^		Gestational weeks	
	(years, mean (SD^a^))		(kg/m^2^, mean (SD))		(week, mean (SD))	
	Boys	Girls	Boys	Girls	Boys	Girls
Normal-weight, nonsmoking mothers (NN)	29.1 (4.0)	29.2 (4.4)	20.9 (1.6)	20.7 (1.6)	38.8 (1.4)	39.2 (1.3)
Normal-weight, smoking mothers (NS)	27.9 (4.8)	27.5 (5.2)	20.8 (1.5)	21.0 (1.8)	38.8 (1.2)	38.5 (2.0)
Underweight, nonsmoking mothers (UN)	27.9 (4.3)	28.5 (4.5)	17.7 (0.7)	17.6 (0.8)	38.8 (1.3)	39.0 (1.5)
Underweight, smoking mothers (US)	26.4 (4.0)	25.9 (5.9)	17.1 (0.9)	17.5 (0.9)	38.9 (1.2)	38.4 (2.2)
Overweight, nonsmoking mothers (ON)	28.5 (4.9)	29.7 (3.7)	27.5 (2.1)	28.2 (2.7)	38.9 (1.7)	39.2 (1.3)
Overweight, smoking mothers (OS)	28.3 (3.4)	26.4 (4.9)	27.8 (2.8)	28.9 (5.6)	39.1 (1.5)	39.3 (1.4)

^a^ Standard deviation;

^b^ Body mass index

**Table 4:** Characteristics of boys and girls in each group.

Variables	Birth order (Mean (SD^a^))		Birth weight (g, Mean (SD))		Infants’ BMI^b^ at birth (kg/m^2^, Mean (SD))	
	Boys	Girls	Boys	Girls	Boys	Girls
Normal-weight and non-smoking mothers (NN)	1.9 (0.9)	1.8 (0.8)	3105.2 (386.5)	3078.7 (378.3)	12.8 (1.1)	12.9 (1.2)
Normal-weight and smoking mothers (NS)	1.7 (0.9)	1.7 (0.9)	2959.0 (343.9)	2893.7 (461.8)	12.5 (1.3)	12.1 (1.3)
Underweight and non-smoking mothers (UN)	1.6 (0.7)	1.7 (0.7)	2979.1 (339.3)	2928.7 (353.8)	12.5 (1.1)	12.4 (1.0)
Underweight and smoking mothers (US)	1.9 (0.9)	1.5 (0.7)	2965.1 (336.7)	2580.7 (725.9)	12.5 (0.9)	11.4 (1.8)
Overweight and non-smoking mothers (ON)	1.7 (0.8)	1.8 (0.7)	3166.9 (438.7)	3139.5 (521.6)	12.9 (1.2)	12.8 (1.6)
Overweight and smoking mothers (OS)	2.2 (0.9)	1.7 (1.0)	2864.0 (378.0)	2898.7 (312.9)	12.1 (0.9)	12.2 (1.1)

a: Standard deviation,

b: Body Mass Index

### BMI z-score trajectories for boys and girls

Solutions for fixed effects of individual growth analyses are presented in Table 5. Overall, Time (p < 0.001), Weight (p < 0.001), the interaction between Time and Smoking (p < 0.001), and the interaction between Time and Weight (p < 0.001) significantly affected boys’ BMI z scores, and Time (p < 0.001), Smoking (p = 0.03), Weight (p < 0.001), and the interaction between Time and Smoking (p = 0.01) significantly affected girls’ BMI z scores.

**Table 5:** Solution for fixed effects according to children’s age and sex and interactions with the combination of maternal weight and smoking status during pregnancy in the Koshu Project, Japan.

Factor		Boys			
		Estimate	Standard Error	t value or F value (Type 3 test)	p
Intercept		-1.15	0.35	-3.30	0.001
Time				**68.38**	**<0.001**
	3 years	1.80	0.38	4.77	<0.001
	5 years	1.88	0.39	4.80	<0.001
	7–8 years	2.26	0.41	5.52	<0.001
	9–10 years	2.31	0.39	5.89	<0.001
Maternal smoking status during pregnancy (Smoking)				**2.55**	**0.1**
	Nonsmoking mothers (NS)	0.67	0.37	1.80	0.07
Maternal pregestational weight status (Weight)				**11.01**	**<0.001**
	Underweight mothers (UW)	0.37	0.44	0.85	0.4
	Normal weight mothers (NW)	0.32	0.38	0.85	0.4
Smoking * Weight				**1.69**	**0.2**
	NS * UW	-0.68	0.47	-1.46	0.1
	NS * NW	-0.38	0.40	-0.95	0.3
Time * Smoking				**5.47**	**<0.001**
	3 years * NS	-0.95	0.40	-2.37	0.02
	5 years * NS	-0.80	0.42	-1.91	0.06
	7–8 years * NS	-1.04	0.43	-2.41	0.02
	9–10 years * NS	-0.78	0.42	-1.86	0.06
Time * Weight				**3.65**	**<0.001**
	3 years * UW	-1.38	0.49	-2.84	0.005
	3 years * NW	-0.37	0.42	-0.89	0.4
	5 years * UW	-1.11	0.51	-2.15	0.03
	5 years * NW	-0.01	0.43	-0.02	0.98
	7–8 years * UW	-1.51	0.51	-2.99	0.003
	7–8 years * NW	-0.62	0.45	-1.37	0.2
	9–10 years * UW	-1.43	0.49	-2.90	0.004
	9–10 years * NW	-0.56	0.44	-1.27	0.2
Time * Smoking * Weight				**1.39**	**0.2**
	3 years * NS * UW	1.11	0.51	2.15	0.03
	3 years * NS * NW	0.18	0.44	0.41	0.7
	5 years * NS * UW	0.58	0.54	1.07	0.3
	5 years * NS * NW	-0.37	0.46	-0.81	0.4
	7–8 years * NS * UW	0.86	0.54	1.60	0.1
	7–8 years * NS * NW	0.08	0.48	0.17	0.9
	9–10 years * NS * UW	0.60	0.52	1.16	0.2
	9–10 years * NS * NW	-0.14	0.46	-0.31	0.8

**Fig 1:**
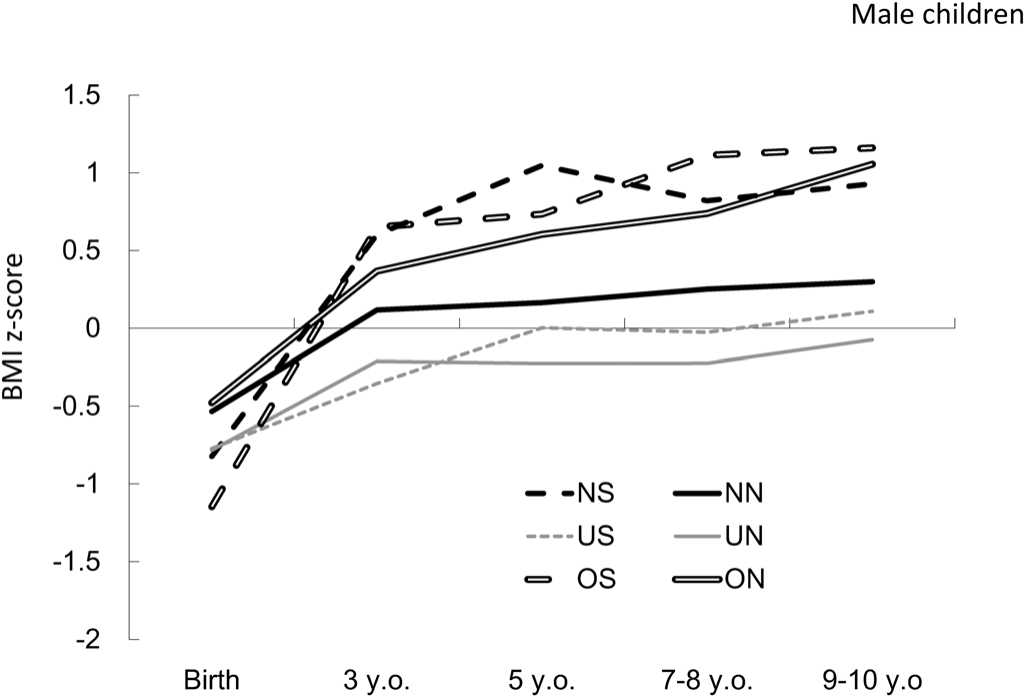
BMI z-score trajectories for boys. BMI z-score trajectories for boys in each combined category according to maternal pregestational weight status and smoking status during pregnancy, calculated using individual growth analysis. BMI: body mass index; NN: normal weight, nonsmoking mother; NS: normal weight, smoking mother; UN: underweight, nonsmoking mother; US: underweight, smoking mother; ON: overweight, nonsmoking mother; OS: overweight, smoking mother; y.o.: years old.

Although boys in the OS group were the leanest at birth, their BMI z scores had increased rapidly by the age of 3 years. In boys in the NS, ON, and OS groups, BMI z scores were more likely to increase between 3 and 9–10 years of age, compared with the other groups. There was a parallel change in the BMI z scores in boys in the NN and UN groups, with lower z scores in the UN group; both groups experienced little change after 3 years of age. BMI z scores in boys in the US group steadily increased by the age of 5 years, after which there was little change (Fig. 1).

**Fig 2:**
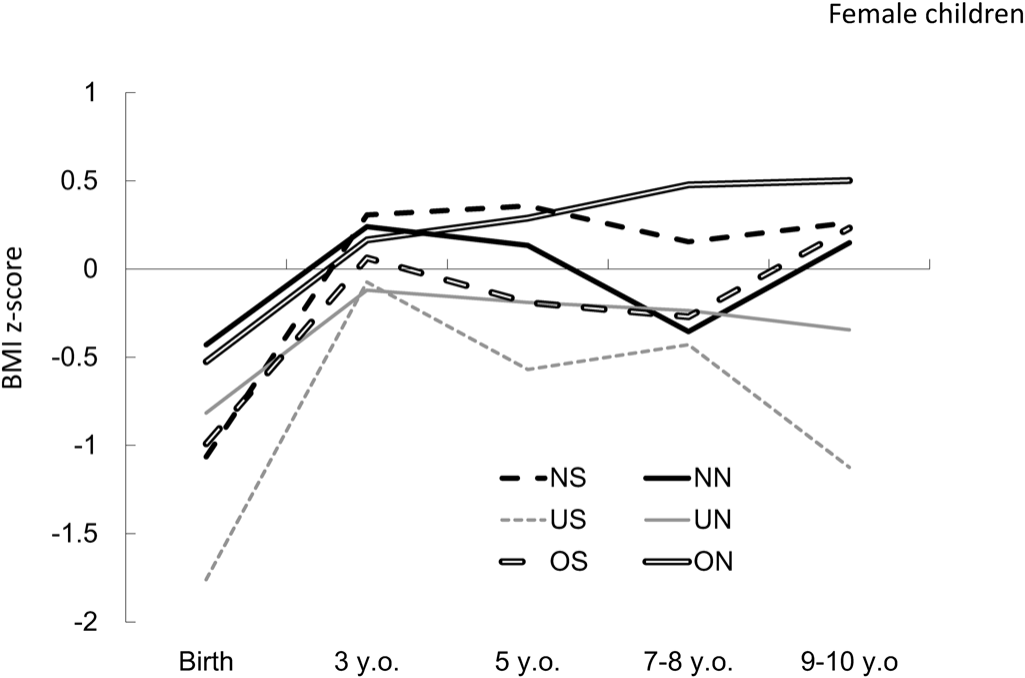
BMI z-score trajectories for girls. BMI z-score trajectories for girls in each combined category according to maternal pregestational weight status and smoking status during pregnancy, calculated using individual growth analysis. BMI: body mass index; NN: normal weight, nonsmoking mother; NS: normal weight, smoking mother; UN: underweight, nonsmoking mother; US: underweight, smoking mother; ON: overweight, nonsmoking mother; OS: overweight, smoking mother; y.o.: years old.

In contrast, BMI z scores decreased in girls in the US group from the age of 3 years. In addition, there was no remarkable difference in BMI z-score trajectories between the other groups between birth and 9–10 years of age. BMI z-scores in girls increased moderately until 3 years old, with no further pronounced change between 3 and 9–10 years of age (Fig. 2).

## Discussion

The results of the present study indicate a graphical sex difference in the effects of the interaction between maternal smoking during pregnancy and pregestational weight status. Particularly, in girls born to underweight and overweight mothers, the effect of maternal smoking during pregnancy could be the opposite of that observed in our previous study, which suggested that BMI was more likely to increase in children of mothers who smoked during pregnancy relative to children of nonsmoking mothers [14]. However, the effect of the interaction between pregestational weight status and maternal smoking during pregnancy on childhood BMI z-score trajectories was not significant.

Smoking rates for expectant mothers who were categorized as either underweight or overweight were higher than those of expectant mothers who were categorized as being of normal weight. In previous studies conducted in Japan and Korea, underweight women were more likely to smoke during pregnancy [24,25]. In addition, the proportion of overweight women classified as nonsmokers during the third trimester of pregnancy was lower than that of women of normal weight; however, the current smoking rate during early pregnancy was higher in overweight women relative to that of women of normal weight [24]. Because the present study used a survey method that differed slightly from that applied in the previous study (for instance, the survey period occurred during pregnancy), comparison of the results is difficult. However, our results were not entirely different from those of the previous study, as the latter suggested that some overweight women who smoked during early pregnancy may have ceased to do so by the third trimester of their pregnancies. Therefore, it is important to examine the association between maternal smoking during pregnancy and pregestational BMI in future studies.

Mean childhood BMI z scores for boys in the NS and OS groups increased rapidly between birth and 3 years of age. The difference between the BMI trajectories measured at each subsequent checkup age for boys born to smoking mothers and children of boys born to nonsmoking mothers approached significance, with higher values for the former, irrespective of maternal pregestational BMI. This finding was consistent with that of our previous study [14]. In particular, the largest difference in BMI z-score trajectories was observed when maternal pregestational BMI was categorized as normal. Because overweight expectant mothers were more likely to bear infants who were large for their gestational ages even though they were smoking during pregnancy, and be obese in childhood, relative to pregnant women of normal weight, the effect of smoking during pregnancy on childhood growth may have been underestimated in overweight women. In addition, the fetal environment could be inappropriate in overweight expectant mothers due to preeclampsia, resulting in excess catch-up growth and overweight in childhood. However, maternal smoking might offer some benefits with respect to preeclampsia [29], which may also have resulted in underestimation of the effect of smoking during pregnancy in overweight pregnant women. In contrast, underweight women were likely to deliver small infants with low birth weights [29]. With this decreased birth weight, maternal smoking during pregnancy could lead to relatively lower risk with respect to perinatal outcomes [30]. Therefore, the effect of smoking during pregnancy on childhood growth was lower in children born to underweight mothers relative to that of children born to normal weight mothers. In addition, there were roughly 2 groups of trajectories. Most of the trajectory values for the OS, NS, and ON groups were higher than 0.5 of the BMI z scores. In contrast, the trajectory values for other groups were within ±0.5 of the BMI z scores. These results suggest that BMI z scores were likely to increase in children in the NS, OS, and ON groups, and the children were more likely to be overweight. Therefore, to prevent childhood obesity, prevention of obesity and smoking in women of reproductive age is important. Moreover, the risk of disease in adulthood may be similar for children in the US group as that for children in the NS and OS groups, although weight status of the child appeared normal in the latter.

There were some differences between girls’ and boys’ trajectories; however, it was impossible to compare these differences statistically. When mothers’ pregestational BMI was categorized as normal, girls’ and boys’ trajectories were similar; however, the difference in trajectories between girls born to smoking and nonsmoking mothers was smaller relative to that observed in boys. Among children born to normal weight mothers, the findings indicating that there was a sex difference in BMI z-score trajectories between children born to smoking and nonsmoking mothers were consistent with previous results [14]. However, the effect of maternal smoking during pregnancy on childhood growth in girls born to underweight or overweight mothers contradicted effects observed for girls born to mothers of normal weight and boys in all groups. There are 2 possible explanations for this observation. In an animal study examining the association between bisphenol A (BPA) and body weight gain, BPA was associated with body weight gain in male offspring; however, female offspring exhibited the opposite effect [31]. Because there are numerous chemicals in cigarette smoke, the findings of this animal study could support our results from a biological perspective. Furthermore, women who subjectively perceived themselves as thin or fat were more likely to smoke [25], and weight concern was a major risk factor for smoking relapse after pregnancy [32]. Because maternal body dissatisfaction can influence their female children [33], it is possible that smoking mothers, particularly those who were underweight or overweight, influenced the weight of their children. However, it is difficult to explain the difference in results between mothers of normal weight and underweight or overweight mothers. Further studies are required to clarify the mechanisms.

This study has some limitations. First, data on maternal smoking during early pregnancy were obtained via a questionnaire. With respect to self-reported smoking in pregnancy, a previous study described relatively low sensitivity; however, specificity was very high [34]. Therefore, in this study, maternal smoking during pregnancy may have been underestimated. In addition, with respect to analysis, we were unable to adjust for participants’ socioeconomic status (SES), which is a potential confounding factor. Further, the numbers of participants in the US and OS groups were small. However, these results were relatively reasonable due to a biologically valid explanation. Moreover, because the participants were all Japanese, it is difficult to generalize the present results to Western countries. However, the results could be important in the prevention of childhood obesity in these countries.

Although our findings should be considered preliminary, the study has certain strengths. To our knowledge, this is the first study to examine the combined effects of pregestational BMI and maternal smoking during pregnancy, both of which are important determinants of childhood growth. Further, the study period spanned approximately 20 years, as children who participated were followed until they reached 9–10 years of age. We were also able obtain the children’s height and body weight data via physical measurements taken during medical checkups conducted in all elementary schools in Koshu city.

In conclusion, there were some differences in childhood growth trajectories according to combinations of maternal pregestational weight status and smoking during pregnancy. Further, sex differences were observed in associations between childhood growth and these combinations. An effective program for the prevention of maternal smoking during pregnancy and overweight prior to pregnancy is necessary to prevent childhood obesity. In addition, the mechanisms underlying the trajectory differences should be clarified in future research. For example, because low SES could be associated with both obesity and smoking [35,36], it is essential to consider this association with respect to the effect of SES on childhood growth.
